# Oncologic Outcomes in a Contemporary “PROTEUS-like” Cohort of High-risk Prostate Cancer Treated with Radical Prostatectomy Without Neoadjuvant Hormonal Therapy

**DOI:** 10.1016/j.euros.2026.08.001

**Published:** 2026-08-24

**Authors:** Can Aydogdu, Benso John, Clara Seizer, Frederik Kolligs, Iason Papadopoulos, Alexander Buchner, Elena K. Berg, Jozefina Casuscelli, Julie Steinestel, Roman Mayr, Matthias Heck, Christian G. Stief, Thilo Westhofen

**Affiliations:** a Department of Urology, LMU University Hospital Munich, Munich, Germany; b Urology, Faculty of Medicine, University of Augsburg, Augsburg, Germany

**Keywords:** High-risk prostate cancer, Radical prostatectomy, Neoadjuvant therapy, PROTEUS trial, Androgen deprivation therapy, Oncologic outcomes

## Abstract

**Background and objective:**

High-risk prostate cancer (PCa) carries substantial risks of recurrence, metastasis, and mortality. Neoadjuvant therapy before radical prostatectomy (RP) remains investigational. The PROTEUS (NCT03767244) trial evaluates perioperative apalutamide + androgen deprivation therapy (ADT) but lacks a surgery-only control cohort. The objective of this study is to characterize oncologic outcomes in patients with PROTEUS-like high-risk PCa undergoing RP without neoadjuvant therapy.

**Methods:**

We performed a retrospective, single-center cohort study of consecutive patients fulfilling PROTEUS high-risk criteria who underwent RP with lymphadenectomy without prior ADT or neoadjuvant treatment. The primary outcome was biochemical recurrence–free survival (BCR-FS). Secondary outcomes included metastasis-free survival (MFS), event-free survival (EFS), and cancer-specific survival (CSS).

**Key findings and limitations:**

Among 1392 patients, median follow-up was 60 mo (95% confidence interval [CI], 56–64); 67% received postoperative radiotherapy. BCR-FS at 36, 60, and 120 mo was 60% (95% CI, 57–64), 44% (95% CI, 40–49), and 22% (95% CI, 17–28), respectively. MFS was 79% (95% CI, 76–83), 71% (95% CI, 66–76), and 56% (95% CI, 47–65), respectively. EFS was 56% (95% CI, 52–60), 40% (95% CI, 36–45), and 18% (95% CI, 13–23), respectively. CSS remained high at 95.5% (95% CI, 94–97), 92% (95% CI, 90–94), and 86% (95% CI, 81–91), respectively. Limitations include the retrospective, single-center design, absence of centralized pathology review, and maturing follow-up.

**Conclusions and clinical implications:**

These findings demonstrate substantial rates of recurrence and metastatic progression despite contemporary management, underscoring the persistent unmet need in this population and providing a rationale for prospective evaluation of perioperative treatment intensification strategies such as PROTEUS.

## Take Home Message

In men meeting strict PROTEUS high-risk criteria, an upfront radical prostatectomy strategy was associated with substantial biochemical recurrence and metastatic progression. These contemporary outcomes provide a real-world benchmark for contextualizing forthcoming PROTEUS results and informing risk-adapted perioperative strategies.

ADVANCING PRACTICE **What does this study add?**

This study reports contemporary real-world oncologic outcomes in men with PROTEUS-defined high-risk prostate cancer managed with an upfront surgery strategy without neoadjuvant hormonal therapy.

**Clinical Relevance**

Because the PROTEUS trial evaluates perioperative systemic therapy without an upfront surgery comparator, these data provide a clinically relevant reference for interpreting outcomes in a similarly defined high-risk population treated with surgery as the initial step of multimodal care. The findings may help contextualize perioperative trial results and inform risk-adapted treatment discussions in high-risk prostate cancer. Associate Editor: Roderick C.N. van den Bergh.

**Patient Summary**

We studied men with high-risk prostate cancer who first underwent surgery and did not receive hormonal treatment before the operation. All patients met the same criteria used in the PROTEUS clinical trial, which is testing whether adding hormonal therapy around the time of surgery improves outcomes. Because the PROTEUS trial does not include a group treated with surgery as the initial approach, our results provide helpful reference information on outcomes after surgery-based care. This may support informed discussions between patients and physicians when considering different treatment options.

## 1 Introduction

Prostate cancer (PCa) is the second most common malignancy among men worldwide [1]. Although most cases are diagnosed at a localized stage, a considerable proportion of cases present with high-risk features such as elevated prostate-specific antigen (PSA) levels, advanced clinical stage, or aggressive histology. These patients carry a substantially increased risk of biochemical recurrence (BCR), metastatic progression, and PCa-specific mortality. Historical radical prostatectomy (RP) series report BCR rates of roughly 45–65% and metastatic progression rates of 15–45% within 5 yr, highlighting the urgent need for more effective perioperative treatment strategies [2].

For men with high-risk disease, current standard management consists of RP with extended pelvic lymph node dissection (PLND) or external beam radiotherapy combined with long-term androgen deprivation therapy (ADT) [3], [4]. Neoadjuvant systemic therapy prior to surgery has not been incorporated into routine practice. Although phase 2 studies, including the ARNEO trial and studies by Taplin et al. [5], demonstrated improved pathological responses with intensified androgen receptor pathway inhibition, a survival benefit has not yet been established [6], [7], [8], [9], [10]. Nonetheless, the biological rationale for perioperative intensification remains strong, particularly given the proven efficacy of apalutamide across multiple advanced PCa disease states [10], [11].

The phase 3 PROTEUS trial (NCT03767244) evaluated perioperative apalutamide + ADT versus ADT + placebo in patients with high-risk or locally advanced PCa undergoing RP. Patients were randomized to 6 mo of apalutamide + ADT or ADT + placebo before RP, followed by an additional 6 mo of treatment after surgery. At 5 yr, metastasis-free survival (MFS) was 78.2% in the apalutamide group versus 73.5% in the placebo group (hazard ratio [HR], 0.80; 95% confidence interval [CI], 0.67–0.96), whereas pathological complete response or minimal residual disease was achieved in 8.9% versus 1.0% of the patients, respectively [12], [13], [14]. Importantly, neither treatment arm reflects current standard clinical practice, as both involve a full year of perioperative systemic therapy rather than immediate surgery alone.

The PROTEUS trial applies a pathology-based definition of high-risk PCa that is more selective than the existing guideline classifications. Instead of relying solely on PSA, grade group, or clinical stage, PROTEUS defines high-risk disease using detailed biopsy characteristics that capture tumor volume and Gleason Score (GS) distribution. This approach identifies a more homogeneous and biologically aggressive subset of localized PCa than the broader high-risk categories defined in European Association of Urology (EAU) and American Urological Association (AUA) guidelines. As a result, the PROTEUS population represents a narrower and more uniform group than the patients who are typically managed with RP in routine practice.

In this study, we evaluate oncologic outcomes in a contemporary cohort of patients who met the principal PROTEUS inclusion criteria but underwent upfront RP without neoadjuvant ADT or androgen receptor signaling inhibition at a high-volume tertiary care center in Germany. Our objective is to characterize the natural history of disease in this PROTEUS-like surgical population.

## 2 Patients and methods

The study was approved by the ethics committee of the Ludwig Maximilian University of Munich (# 20-1022) and conducted in accordance with the principles of the Declaration of Helsinki. The data cutoff for this analysis was September 24, 2025, and all analyses were performed from October 2025 to November 2025.

### 2.1 Study population

Patients were eligible for inclusion if they had histologically confirmed prostate adenocarcinoma and had undergone RP with PLND between January 2009 and January 2024. *High-risk disease* was defined in accordance with the PROTEUS trial criteria as Gleason Grade Group ≥3 (GS ≥4+3), together with at least one of the following: ≥6 systematic biopsy cores containing GS 4+3, including at least one core with GS 8 (a); ≥3 such cores in combination with a PSA concentration ≥20 ng/ml and at least one core with GS 8 (b); GS ≥9 in any systematic or targeted core (c); or ≥2 systematic or targeted cores demonstrating continuous GS ≥8 with >80% tumor involvement (d). More than one category could apply to an individual patient. For exploratory subgroup analyses, patients were assigned the highest applicable category (d > c > b > a).

Only patients with complete baseline clinical information (PSA, GS, clinical stage, and biopsy characteristics) and a minimum follow-up duration of 12 mo (unless recurrence or death occurred earlier) were included. Patients were excluded if they had distant metastases (M1), had received any systemic or local treatment prior to RP, did not meet the PROTEUS eligibility criteria (*n* = 3734), had insufficient baseline information to determine PROTEUS eligibility because of missing biopsy or staging data (*n* = 432), or had missing follow-up or a follow-up duration of <12 mo (*n* = 134). No meaningful temporal differences in missing baseline or pathological data were observed across the study period (Supplementary Fig. 1). Over the study period, diagnostic workup evolved in line with prevailing standards of care: between 2009 and 2014, staging relied predominantly on conventional imaging with computed tomography (CT) and bone scintigraphy and systematic transrectal biopsy. From ∼2015 onward, increasing availability of multiparametric MRI led to gradual adoption of MRI-targeted biopsy. In later years (from around 2020), selective use of Prostate-Specific Membrane Antigen Positron Emission Tomography (PSMA-PET) was introduced for staging and recurrence assessment.

### 2.2 Definitions of end points and outcomes

The primary end point was *BCR-free survival (BCR-FS)*, defined as the time from RP to the first postoperative PSA value of ≥0.2 ng/ml confirmed by a second consecutive rise, or the initiation of salvage therapy, whichever occurred first, with censoring at last follow-up or death.

Secondary end points included MFS, event-free survival (EFS), and cancer-specific survival (CSS). *MFS* was defined as the time from surgery to radiologically confirmed distant metastasis or death of any cause; *EFS* as the time from surgery to BCR, radiographically confirmed metastases or death of any cause; and *CSS* as the time from surgery to death attributable to PCa.

*PSA persistence* was defined as a detectable serum PSA level 6–12 wk after RP.

### 2.3 Statistical analysis

Statistical analyses and reporting and interpretation of the results were conducted according to Guidelines for Reporting of Statistics for Clinical Research in Urology [15]. Descriptive statistics were reported as median values with interquartile ranges or as proportions, as appropriate. Time-to-event outcomes were estimated using the Kaplan-Meier method. Univariable Cox proportional hazards models were used to estimate HRs with 95% CIs for selected binary variables. The proportional hazards assumption was assessed using Schoenfeld residuals and was met for all Cox models. Landmark estimates were provided at 36, 60, and 120 mo following RP, including 95% CIs. Median follow-up time was calculated using the reverse Kaplan-Meier method. All statistical tests were two sided, and *p* values ≤0.05 were considered statistically significant. Statistical analyses were conducted using MedCalc Statistical Software version 20.011 (MedCalc Software, Ostend, Belgium).

## 3 Results

### 3.1 Baseline characteristics of the study population

Baseline characteristics of the 1392 patients who met PROTEUS high-risk criteria are summarized in Table 1. Median follow-up was 60 mo (95% CI, 56–64). The cohort was characterized by a median age of 68 yr and a median preoperative PSA of 11.4 ng/ml. High-grade disease was predominant, with GS 9–10 in 62%, and patients showed a substantial tumor burden on biopsy. Most had locally advanced pathology at RP, with pT3a/b tumors in 76% and pathologic lymph node involvement in 28%. Positive surgical margins were observed in 20% of the cases. A robot-assisted approach was used in 29% of cases, and 67% of patients subsequently received postoperative radiotherapy. Of these, 24% received adjuvant, and 43% received salvage radiotherapy. Baseline characteristics stratified by treatment era are summarized in Supplementary Table 1.

**Table 1:** Baseline characteristics

Variable	Result
**Patients (*n*)**	1392
**Age (yr), median (IQR)**	68 (63–73)
**BMI (kg/m^2^), median (IQR)**	26.9 (24.3–29.1)
**Preoperative PSA (ng/ml), median (IQR)**	11.4 (6.9–24.6)
**Prostate volume (ml), median (IQR)**	56 (45–71)
**CCI, *n* (%)**	
1	18 (1)
2	195 (14)
3	557 (40)
4	572 (41)
5	50 (4)
**Biopsy GS, *n* (%)**	
8	536 (38)
9	752 (54)
10	104 (8)
**Number of biopsy cores, median (IQR)**	12 (11–14)
**Number of positive biopsy cores, median (IQR)**	8 (6–10)
**Percentage of cancer per core, median (IQR)**	47.2 (30.0–65.4)
**Biopsy technique, *n* (%)**	
Transrectal	1193 (86)
Transperineal	199 (14)
**Prostate MRI, *n* (%)**	494 (36)
**MRI fusion biopsy, *n* (%)**	477 (34)
**pT stage, *n* (%)**	
pT2a	19 (1)
pT2b	8 (1)
pT2c	276 (20)
pT3a	347 (25)
pT3b	711 (51)
pT4	31 (2)
**Positive surgical margin, *n* (%)**	272 (20)
**Pathologic lymph node involvement, *n* (%)**	383 (28)
**Robot-assisted RP, *n* (%)**	406 (29)
**Postoperative RT, *n* (%)**	937 (67)
Adjuvant RT, *n* (%)	331 (24)
Salvage RT, n (%)	606 (43)

BMI = body mass index; CCI = Charlson Comorbidity Index; GS = Gleason score; IQR = interquartile range; MRI = Magnetic resonance imaging; PSA = prostate-specific antigen; pT = pathologic T stage; RP = radical prostatectomy; RT = radiotherapy.

### 3.2 Survival outcomes

**Fig. 1:**
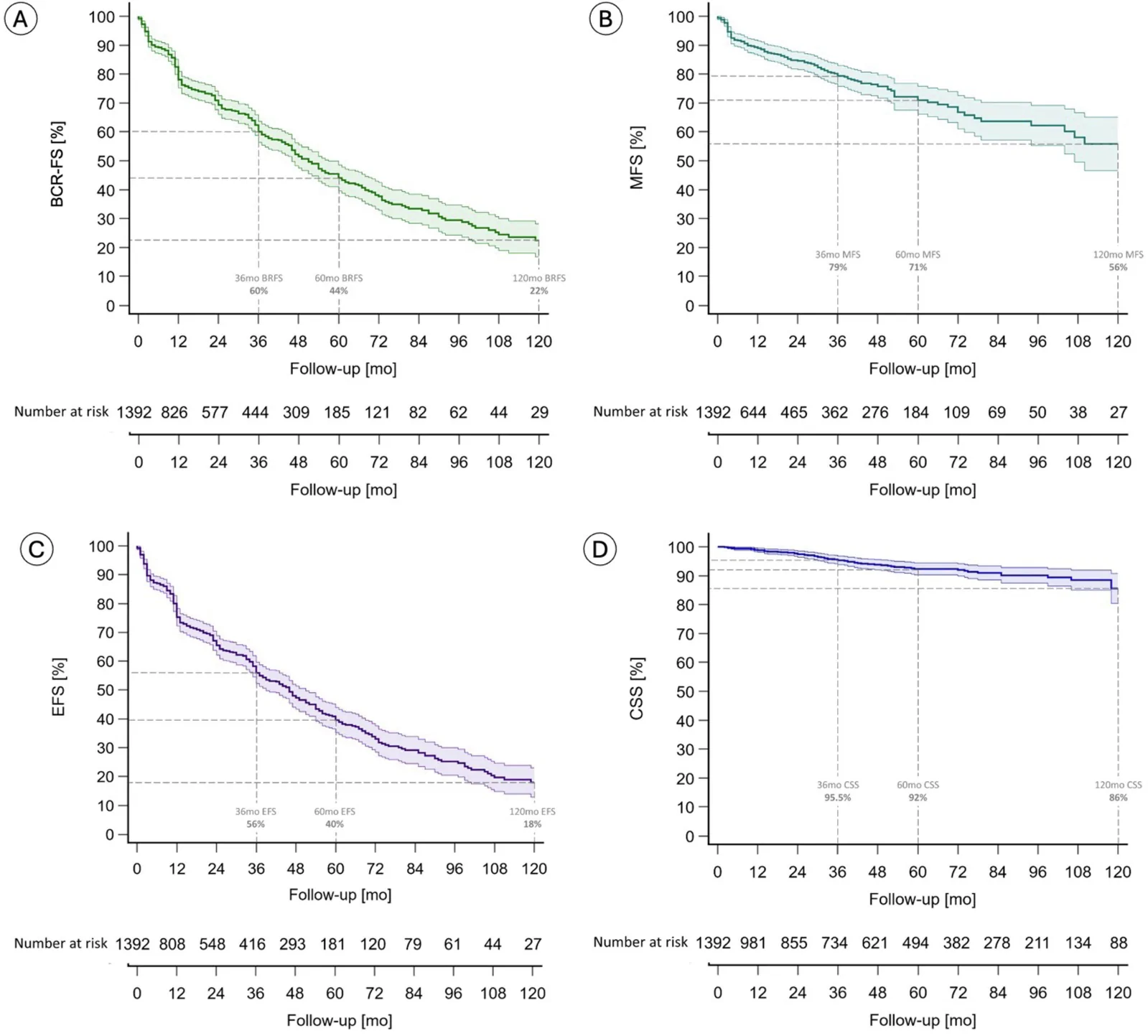
Kaplan-Meier analyses of oncologic outcomes after radical prostatectomy with 36-, 60-, and 120-mo landmarks. (A) BCR-FS, (B) MFS, (C) EFS, and (D) CSS. Shaded areas represent 95% confidence intervals, and numbers at risk are displayed beneath each panel. Together, these curves illustrate the temporal patterns of disease control and cancer-related mortality in the study cohort. BCR-FS = biochemical recurrence–free survival; CSS = cancer-specific survival; EFS = event-free survival; MFS = metastasis-free survival.

At the time of analysis, 671 patients had experienced biochemical failure. At the 36-, 60-, and 120-mo landmarks, BCR-FS was 60% (95% CI, 57–64), 44% (95% CI, 40–49), and 22% (95% CI, 17–28), respectively (Fig. 1A). Patients with pathologic lymph node involvement (pN1) had a significantly higher risk of BCR than patients with pN0 (HR, 1.34; 95% CI, 1.09–1.65; *p* = 0.005).

**Fig. 2:**
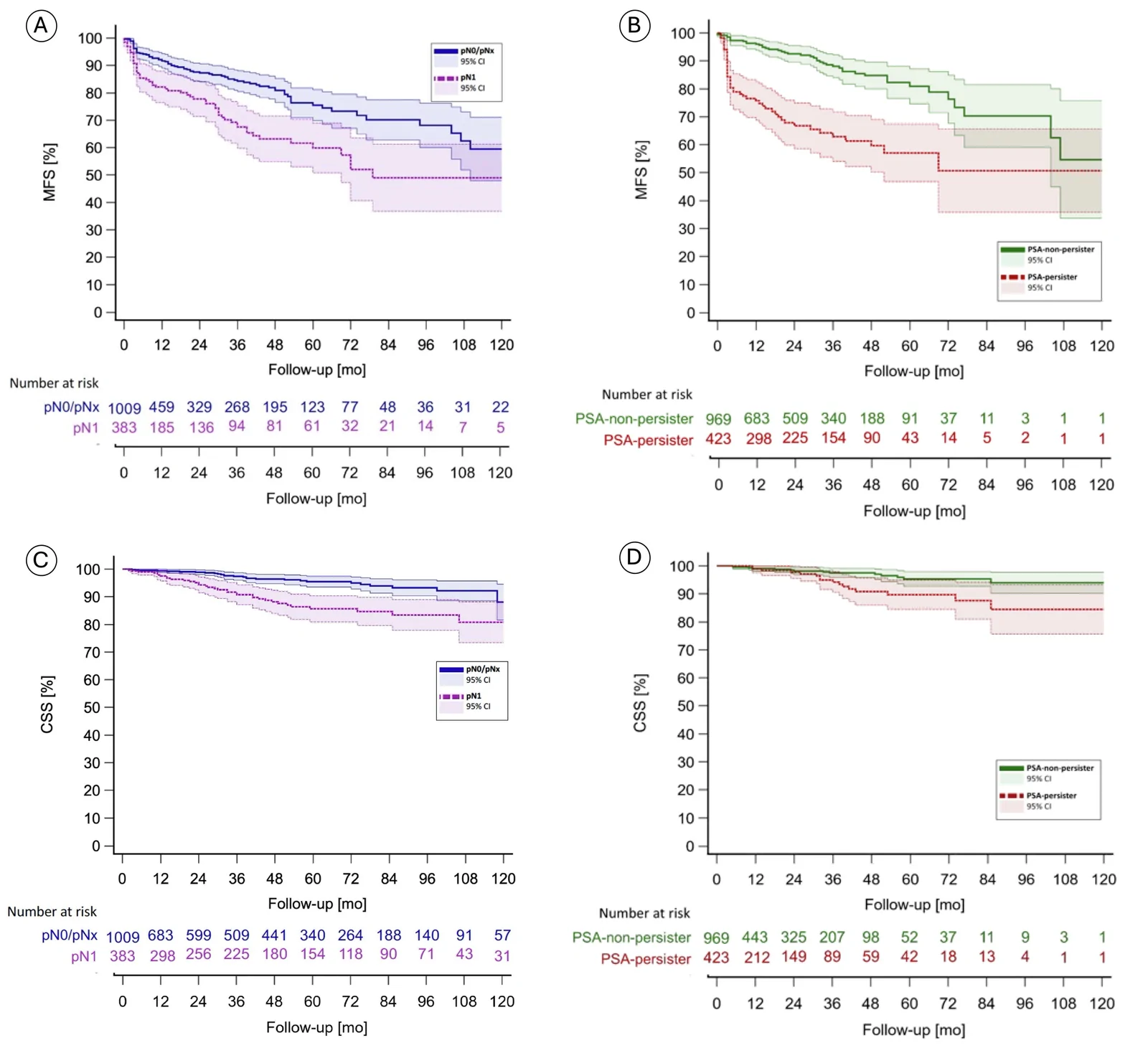
Kaplan-Meier survival stratified by pathologic nodal status and postoperative PSA persistence. (A) MFS stratified by pathologic nodal status (pN0 vs pN1). (B) MFS stratified by postoperative PSA persistence. (C) CSS stratified by pathologic nodal status (pN0 vs pN1). (D) CSS stratified by postoperative PSA persistence. Numbers at risk for each group are shown below the corresponding survival curves. CSS = cancer-specific survival; MFS = metastasis-free survival; PSA = prostate-specific antigen.

A total of 290 patients had experienced distant metastasis at the time of analysis. MFS at 36, 60, and 120 mo was 79% (95% CI, 76–83), 71% (95% CI, 66–76), and 56% (95% CI, 47–65), respectively (Fig. 1B). Patients with pN1 had a significantly higher risk of metastatic progression than patients with pN0 (HR, 1.96; 95% CI, 1.41–2.73; *p* < 0.001), with lower MFS at 36, 60, and 120 mo (68% vs 84%, 60% vs 76%, and 49% vs 60%, respectively). Similarly, PSA persistence was associated with inferior MFS at 36, 60, and 120 mo (63% vs 88%, 57% vs 81%, and 51% vs 55%, respectively; HR, 3.26; 95% CI, 2.19–4.85; *p* < 0.001; Fig. 2A and 2C).

At the time of analysis, 93 deaths from PCa occurred within the cohort. CSS was 95.5% (95% CI, 94–97), 92% (95% CI, 90–94), and 86% (95% CI, 81–91) at 36, 60, and 120 mo, respectively (Fig. 1D). CSS was lower in patients with pN1 at 36, 60, and 120 mo (92% vs 97%, 86% vs 96%, and 81% vs 88%, respectively; HR, 2.96; 95% CI, 1.99–4.38; *p* < 0.001). Likewise, PSA persistence was associated with inferior CSS at 36, 60, and 120 mo (95% vs 98%, 90% vs 95%, and 84% vs 94%, respectively; HR, 2.31; 95% CI, 1.27–4.18; *p* = 0.006; Fig. 2B and 2D).

At the time of analysis, 739 patients met an EFS end point. Corresponding EFS at 36, 60, and 120 mo was 56% (95% CI, 52–60), 40% (95% CI, 36–45), and 18% (95% CI, 13–23), respectively (Fig. 1C).

**Fig. 3:**
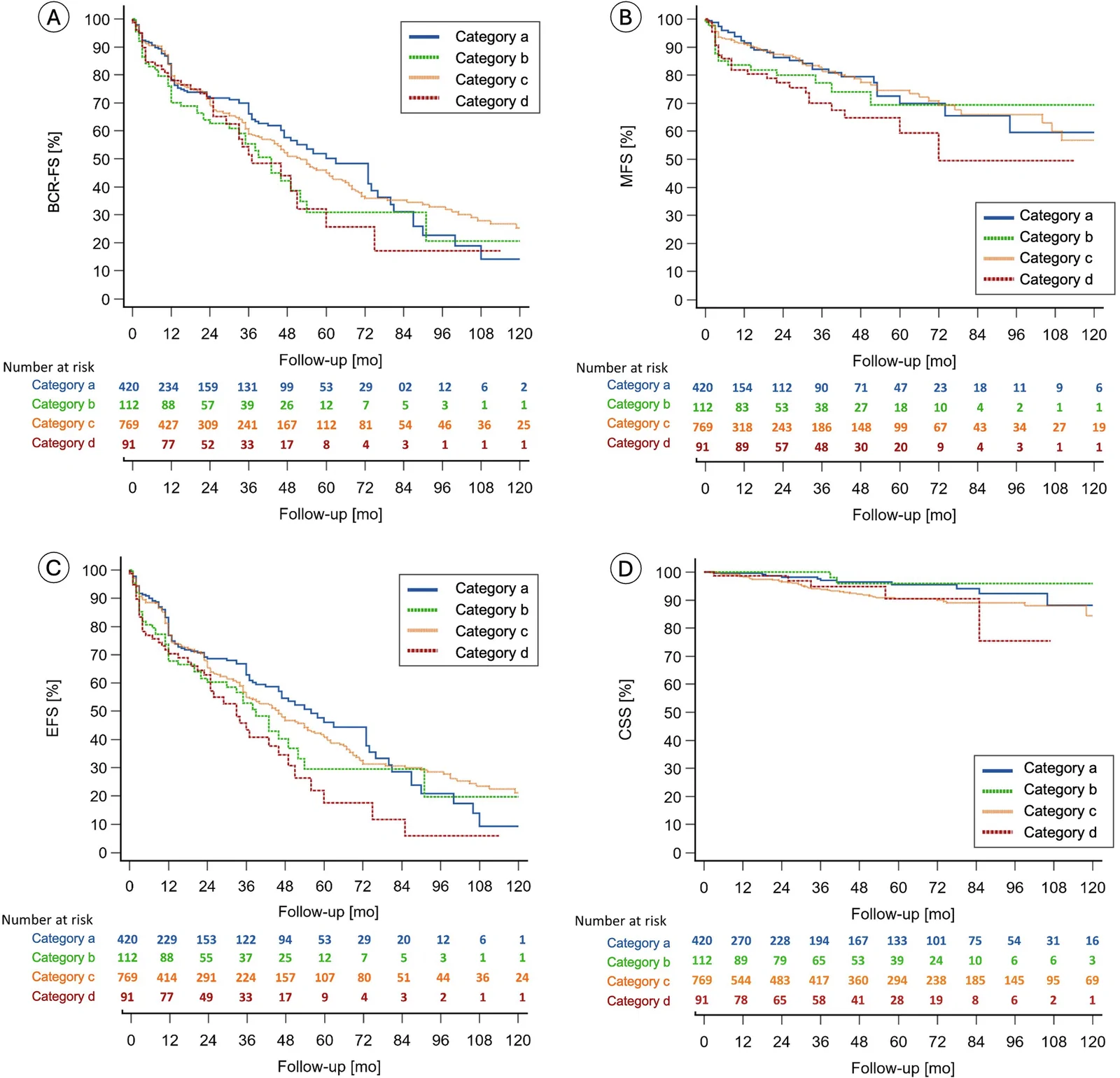
Kaplan-Meier survival analyses by PROTEUS risk category. (A) Kaplan-Meier curves for BCR-FS. (B) Kaplan-Meier curves for MFS. (C) Kaplan-Meier curves for EFS. (D) Kaplan-Meier curves for CSS. Numbers at risk at each time point are shown below the plots. Risk categories were assigned according to PROTEUS high-risk inclusion criteria; when patients met criteria for multiple categories, the highest applicable category (d > c > b > a) was used. BCR-FS = biochemical recurrence–free survival; CSS = cancer-specific survival; EFS = event-free survival; MFS = metastasis-free survival.

In an exploratory analysis stratified by PROTEUS risk categories from a to d, outcome patterns were broadly similar across groups, and no formal statistical testing was performed (Fig. 3A–D).

## 4 Discussion

In this study, we report a large cohort of 1392 men with high-risk PCa who met the strict inclusion criteria of the PROTEUS trial and thus serve as a contemporary reference for oncologic outcomes after upfront RP in this narrowly defined population. Since completion of our analysis, the phase 3 PROTEUS trial demonstrated significantly improved oncologic outcomes with perioperative apalutamide + ADT, including a 5-yr MFS of 78.2% compared with 73.5% for ADT + placebo (HR, 0.80; 95% CI, 0.67–0.96). By comparison, the 5-yr MFS in our contemporary surgical cohort was 71%. Importantly, the PROTEUS investigators also report that a complementary substudy comparing perioperative therapy with RP alone is ongoing. Our findings therefore provide benchmark oncologic outcomes for patients treated with upfront surgery and may help contextualize the results of this complementary analysis. After applying the PROTEUS pathological selection criteria, this rigorously defined cohort showed unfavorable long-term oncologic outcomes. BCR-FS was 60%, 44%, and 22% at 3, 5, and 10 yr, respectively, after RP. MFS was 79%, 71%, and 56%, and EFS was 56%, 40%, and 18% at the corresponding landmarks. CSS remained high but declined to 96%, 92%, and 86%, respectively. The reported outcomes reflect an upfront surgery strategy followed by standard-of-care adjuvant or salvage therapy, consistent with contemporary multimodal management of high-risk PCa. Although surgery enables postoperative risk stratification and selective treatment escalation, the observed recurrence and metastasis rates highlight the persistent unmet need in this population and support continued evaluation of perioperative treatment intensification strategies.

Although differences in risk definitions, treatment eras, and follow-up limit direct comparability, several definitions of high-risk PCa have been validated in real-world cohorts. Across large contemporary surgical series using D’Amico or similar high-risk criteria, the 5-yr BCR-FS ranged from 47.7% in overall high-risk cohorts to 35.4% among men with GS ≥8, whereas 10-yr cancer-specific mortality ranged from 5.1% to 12.8% [16], [17]. Outcomes were consistently worse among patients harboring multiple adverse risk factors [16], [17]. Similarly, Abdollah et al [18] demonstrated marked heterogeneity within high-risk disease, reporting 10-yr freedom from local or distant recurrence ranging from 87.4% in the overall high-risk cohort to 54.6% among men with both GS ≥8 and PSA ≥10 ng/ml. In a contemporary cohort of 16 746 men, Falagario et al [19] reported BCRs exceeding 30% after RP, whereas PCa-specific mortality remained <5%, highlighting the discordance between BCR and lethal disease in the era of multimodal management.

A definition most closely aligned with the PROTEUS high-risk criteria was described by Sundi et al [20]. They defined very high-risk disease by the presence of primary pattern 5, ≥5 biopsy cores with GS ≥8, or multiple National Comprehensive Cancer Network (NCCN) high-risk features. Among 144 men who met these criteria, 10-yr BCR-FS was 21%, MFS was 37%, and CSS was 62%. Our cohort shows comparable 10-yr BCR-free and metastasis-free outcomes. The lower CSS reported by Sundi et al [20] likely reflects the more limited availability of effective systemic salvage options at the time of their 2014 analysis.

Data from randomized and prospective neoadjuvant studies indicate that short-term ADT, chemohormonal therapy, or androgen receptor pathway inhibition before RP can improve pathological outcomes but have not consistently translated into durable oncologic benefits [9], [5], [21], [22], [23], [24]. Although Ravi et al [25] reported improved BCR-FS and MFS with novel hormonal agent-based neoadjuvant therapy compared with upfront RP, robust survival advantages remain unproven.

Our study has several limitations. First, the 15-yr inclusion period spans a phase of rapid evolution in systemic therapy for advanced PCa. Key developments include the introduction of docetaxel, novel hormonal agents, PSMA radioligand therapy, and Poly-ADP Ribose Polymerase (PARP) inhibitors. Although these treatments have limited effect on BCR or MFS, they can substantially influence CSS, which complicates comparisons across treatment eras. Second, the retrospective design introduces potential selection bias. Analyses were performed using a complete-case approach, and the potential for additional bias because of exclusion of incomplete cases cannot be excluded. Third, this is a single-center academic cohort with referral-based characteristics and high-volume surgeons. Treatment patterns, use of multimodal therapy, and surgical expertise may differ in community settings, which limits generalizability. Oncologic outcomes cannot be attributed to surgery alone because adjuvant and salvage radiotherapy reflect contemporary standard-of-care multimodal management; excluding these patients would have preferentially removed those at highest risk and introduced selection bias. The high proportion of patients requiring postoperative radiotherapy further underscores the aggressive nature of this PROTEUS-like population.

Fourth, imaging standards evolved considerably during the study period. Conventional CT and bone scan staging gradually transitioned to PSMA-PET/CT, which has higher sensitivity for detecting oligometastatic disease, particularly because patients who would previously have been classified as having localized high-risk disease may now be identified as having occult metastatic disease. Notably, PROTEUS eligibility was originally established using conventional imaging-based staging criteria. Similarly, the increasing use of MRI-targeted biopsy may have improved detection of high-grade disease and influenced risk classification over time. This shift may have contributed to stage migration and altered downstream management decisions. Fifth, although all pathological assessments were performed at our institution, specimens were not rereviewed. Given changes in Gleason grading over time, especially after the 2005 and 2014 International Society of Urological Pathology (ISUP) revisions, some temporal variation in risk classification cannot be entirely excluded. Therefore, this cohort represents a real-world approximation of the PROTEUS population.

Finally, all patients in this study underwent RP. Increasing evidence supports surgery as a valid option for men with high-risk disease, but any risk stratification approach, including the PROTEUS definition, must ultimately be interpretable across different treatment modalities.

Despite these limitations, the large cohort size and long follow-up strengthen the robustness of our findings and support the use of this dataset as a contemporary benchmark for oncologic outcomes in patients meeting the PROTEUS high-risk criteria. Additional multicenter retrospective studies are warranted to confirm these results and further contextualize perioperative trials within contemporary standards of care.

## 5 Conclusions

In this contemporary cohort meeting the PROTEUS high-risk criteria, a surgical-first management strategy resulted in considerable rates of BCR and metastatic progression, underscoring the biological aggressiveness of this narrowly defined population. These findings provide a clinically relevant benchmark for comparison with perioperative treatment strategies and may help contextualize the results of the ongoing complementary PROTEUS substudy evaluating RP alone.

  ***Author contributions***: Can Aydogdu had full access to all the data in the study and takes responsibility for the integrity of the data and the accuracy of the data analysis.

  *Study concept and design*: Aydogdu, Westhofen.

*Acquisition of data*: Aydogdu, John, Seizer, Kolligs, Papadopoulus, Buchner.

*Analysis and interpretation of data*: Aydogdu, Westhofen.

*Drafting of the manuscript*: Aydogdu.

*Critical revision of the manuscript for important intellectual content*: John, Seizer, Kolligs, Papadopoulus, Buchner, Berg, Casuscelli, Steinestel, Mayr, Heck, Stief, Westhofen.

*Statistical analysis*: Westhofen.

*Obtaining funding*: None.

*Administrative, technical, or material support*: Westhofen.

*Supervision*: Westhofen.

*Other* (specify): None.

  ***Financial disclosures:*** Can Aydogdu certifies that all conflicts of interest, including specific financial interests and relationships and affiliations relevant to the subject matter or materials discussed in the manuscript (eg, employment/affiliation, grants or funding, consultancies, honoraria, stock ownership or options, expert testimony, royalties, or patents filed, received, or pending), are the following: None.

  ***Funding/Support and role of the sponsor*:** None.

  ***Informed consent statement*:** This study was approved by the ethics committee of the Medical Faculty of Ludwig Maximilians University of Munich, and informed consent was waived.

  ***Data availability statement*:** Data are available for bona fide researchers who request them from the authors.
