# Supplementary material for: Oncologic Outcomes in a Contemporary “PROTEUS-like” Cohort of High-risk Prostate Cancer Treated with Radical Prostatectomy Without Neoadjuvant Hormonal Therapy
Source: Eur Urol Open Sci. 2026 Aug 24;92:10–8. doi: 10.1016/j.euros.2026.08.001 (PMC13543131; doi:10.1016/j.euros.2026.08.001)
Supplement: Supplementary file 1 [file mmc1.docx]

**Supplementary material**

**Figure S1. Study flow diagram.** Flow diagram illustrating patient selection from 5,692 patients who underwent radical prostatectomy (RP) with pelvic lymph node dissection (PLND) between January 2009 and January 2024. Patients not meeting the PROTEUS criteria or with insufficient data to determine PROTEUS eligibility were excluded. Patients meeting the PROTEUS inclusion criteria with missing follow-up or follow-up <1 year were subsequently excluded. The final study cohort comprised 1,392 patients. Numbers of excluded patients are stratified by time period. RP = radical prostatectomy; PLND = pelvic lymph node dissection.

| **Variable** | **2009–2014** | | **2015–2019** | | **2020–2024** | |  |
| --- | --- | --- | --- | --- | --- | --- | --- |
| **No. of patients, n(%)** | 128 (9) | | 282 (20) | | 982 (71) | |  |
| **Median age, yr (IQR)** | 68 (63–71) | | 68 (61–72) | | 69 (63–74) | |  |
| **Median BMI, kg/m^2^ (IQR)** | 26.8 (24.9–29) | | 26.6 (24.7–28.7) | | 26.6 (24.2–29.1) | |  |
| **Median preoperative PSA, ng/mL (IQR)** | 11 (6.6–27.5) | | 10 (6.5–26.3) | | 11.9 (7.1–23.7) | |  |
| **Median prostate volume, ml (IQR)** | 52.5 (42–67.3) | | 54 (44–68) | | 56 (45–71) | |  |
| **Charlson comorbidity index, n (%)** |  | |  | |  | |  |
| 1 | 2 (1.6) | | 3 (1.1) | | 13 (1.3) | |  |
| 2 | 17 (13.3) | | 52 (17.8) | | 126 (12.7) | |  |
| 3 | 66 (51.6) | | 115 (40.9) | | 376 (38.3) | |  |
| 4 | 39 (30.5) | | 108 (38.4) | | 425 (43.3) | |  |
| 5 | 4 (3.1) | | 4 (1.8) | | 42 (4.4) | |  |
| **Biopsy GS, n (%)** |  | |  | |  | |  |
| 8 | 30 (23) | | 104 (37) | | 402 (41) | |  |
| 9 | 88 (69) | | 164 (58) | | 500 (51) | |  |
| 10 | 10 (8) | | 14 (5) | | 80 (8) | |  |
| **Median number of biopsy cores (IQR)** | 11 (8–12) | | 12 (10–12) | | 12 (12–14) | |  |
| **Median number of positive biopsy cores (IQR)** | | 8 (6–10) | | 8 (5.5–10) | | 8 (6–10) | |
| **Median percentage of cancer per core (IQR)** | 48.6 (38.1–85.9) | | 42.9 (32–82) | | 48 (44.2–60.3) | |  |
| **Biopsy technique, n (%)** |  | |  | |  | |  |
| Transrectal | 128 (100) | | 281 (99.6) | | 784 (80) | |  |
| Transperineal | 0 (0) | | 1 (0.4) | | 198 (20) | |  |
| **Prostate MRI, n (%)** | 0 (0) | | 38 (14) | | 456 (46) | |  |
| **MRI fusion biopsy, n (%)** | 0 (0) | | 38 (14) | | 439 (45) | |  |
| **pT stage, n (%)** |  | |  | |  | |  |
| pT2a | 1 (1) | | 1 (0.5) | | 17 (2) | |  |
| pT2b | 0 (0) | | 1 (0.5) | | 7 (1) | |  |
| pT2c | 17 (13) | | 50 (18) | | 209 (21) | |  |
| pT3a | 27 (21) | | 73 (26) | | 247 (25) | |  |
| pT3b | 79 (62) | | 147 (52) | | 485 (49) | |  |
| pT4 | 4 (3) | | 10 (3) | | 17 (2) | |  |
| **Positive surgical margin, n (%)** | 28 (22) | | 62 (22) | | 182 (19) | |  |
| **Lymph node involvement, n (%)** | 44 (34) | | 94 (33) | | 245 (25) | |  |
| **Robot-assisted RP, n (%)** | 0 (0) | | 43 (15) | | 363 (37) | |  |
| **Postoperative RT, n (%)** | 107 (84) | | 241 (86) | | 589 (60) | |  |
| adjuvant RT, n (%) | 30 (23) | | 44 (16) | | 190 (19) | |  |
| salvage RT, n (%) | 77 (60) | | 197 (70) | | 399 (41) | |  |

**Supplementary Table S1: Baseline clinical, diagnostic, and pathological characteristics stratified by treatment era.**
